# The Implications of Changing Age and Weight at Slaughter of Heavy Pigs on Carcass and Green Ham Quality Traits

**DOI:** 10.3390/ani11082447

**Published:** 2021-08-20

**Authors:** Isaac Hyeladi Malgwi, Luigi Gallo, Veronika Halas, Valentina Bonfatti, Giuseppe Carcò, Celio Paolo Sasso, Paolo Carnier, Stefano Schiavon

**Affiliations:** 1 Department of Agronomy, Food, Natural Resources, Animals and Environment (DAFNAE), University of Padova, Viale dell’ Università 16, 35020 Legnaro, Padova, Italy; luigi.gallo@unipd.it (L.G.); giuseppe.carco@live.it (G.C.); celiopaolo.sasso@gmail.com (C.P.S.); stefano.schiavon@unipd.it (S.S.); 2 Department of Farm Animal Nutrition, Hungarian University of Agriculture and Life Sciences (MATE), Kaposvár Campus, Guba Sándor Utca 40, H-7400 Kaposvár, Hungary; Halas.Veronika@uni-mate.hu; 3 Department of Comparative Biomedicine and Food Science (BCA), University of Padova, Agripolis, Viale dell’Università 16, 35020 Legnaro, Padova, Italy; valentina.bonfatti@unipd.it (V.B.); paolo.carnier@unipd.it (P.C.)

**Keywords:** carcass quality, dry-cured ham, growth performance, pigs, slaughter age, slaughter weight

## Abstract

**Simple Summary:**

Conventional rearing systems for heavy pigs intended for Italian dry-cured ham production require pigs to be slaughtered at 160 ± 16 kg and a minimum age of 9 months. With the current animal genetic trends providing progressively leaner animals, the conventional rearing system fails to provide pigs with optimal characteristics for the dry-cured ham industry. In this research, new combinations of age and weight at slaughter were explored, using different feeding conditions, as possible alternative rearing strategies for heavy pigs. Such alternative rearing strategies aimed to manipulate the growth rate of pigs, first allowing them to reach 160 ± 16 kg slaughter weight at a younger age; second, allowing pigs to maximize their slaughter weight at 9 months of age; and third, inducing slow growth in the pigs to reach the 160 ± 16 kg body weight at an older age. The first two strategies were the most promising alternatives as they improved the rate of gain, feed efficiency, and ham adiposity of the pigs. While the first strategy was the most economically convenient, the second produced the hams with the highest quality.

**Abstract:**

Italian dry-cured ham production requires pigs to be slaughtered at 160 ± 16 kg at 9 months of age (control, C). The study explored three alternatives, based on different feeding conditions: (1) allowing pigs to express their growth potential by letting them reach 160 ± 16 kg slaughter weight (SW) at younger slaughter age (SA) (younger Age, YA); (2) allowing pigs to express their growth potential by maximizing their SW at 9 months SA (greater weight, GW); (3) increasing the SA required to reach 160 ± 16 kg SW (older age, OA). Pigs (336 C21 Goland, 95 kg initial body weight) were slaughtered on average at 257, 230, 257, and 273 d SA and 172.7, 172.3, 192.9, and 169.3 SW kg for the four treatments, respectively. C pigs had an average daily gain (ADG) of 715 g/d and feed efficiency (FE) of 0.265 (gain to feed). Compared to C, YA pigs had higher ADG (+32%), FE (+7.5%), and better ham adiposity; GW pigs had higher carcass weight (+12%), ADG (+25%), trimmed ham weight (+10.9%), and better ham adiposity. OA treatment affected ADG (−16.4%), FE (−16.6%), and trimmed ham weight (−3.6%). YA and GW could be promising alternatives to C as they improved FE and ham quality traits.

## 1. Introduction

Setting target slaughter weight (SW) and slaughter age (SA) is a management decision that impacts the productivity and profitability of pig production [1]. Over the past decades, the availability of lean pig lines has led to an increase in the SW in several countries, to minimize the cost of production per pig [1]. Globally, in the dry-cured ham pig production chain, heavy SW and advanced SA are required to ensure adequate ham size, ham fat covering depth, and lean tissue maturity [2,3]. Most often, SW and SA are mandatory criteria for quality assurance of hams and other pig products [4].

Product specifications of Italian Protected Designation of Origin (PDO) dry-cured hams set the minimum SA and SW to 9 months and 160 ± 16 kg, respectively [5,6]. To fulfil these requirements, the average daily gain (ADG) from birth to slaughter must be constrained to 0.60–0.70 kg/d. This leads to the adoption of restricted feeding strategies based on medium protein diets [7]. However, the supply of increasingly lean pig lines results in a growing proportion of hams that do not meet the industry quality standards [6,8]. An adjustment in SW and SA can result in higher carcass fatness and better ham characteristics [3,9]. Currently, the guidelines of the existing PDO product specifications are undergoing revision by the ham consortia. Given possible changes in the requirements for Italian heavy pig farming, valid alternatives to the current rearing strategy should be investigated. In addition, it can be observed that due to animal welfare issues, feeding strategies that allow the pigs to eat ad libitum rather than restricted might be more appreciated by the consumers.

Different combinations of age and weight at slaughter can be attained by manipulating energy and dietary nutrient supplies [10,11], and this may affect not only raw ham properties [3] but also growth performance and carcass traits of pigs. Therefore, this study was arranged to compare the current conventional production system to the following three alternative feeding and rearing strategies, representing different combinations of age and weight at slaughter: (i) allowing pigs to reach the conventional SW of 160 ± 16 kg at a younger age; (ii) allowing pigs to maximize their SW at the conventional SA of 9 months of age; and (iii) increasing the time required by the pigs to reach the conventional SW of 160 ± 16 kg SW. Compared with the conventional system, both the first and the second strategies imply an increased growth rate obtained increasing energy and dietary nutrient supplies. Conversely, the third strategy implies a reduction in the ADG, attainable through a reduced energy and protein supply, as this will stimulate a greater fat deposition at the expense of the lean growth [11]. There are limited reports regarding the effect of an increased SW or SA in heavy pigs [2,9]. To our knowledge, prior studies have not considered such factors nor evaluated the consequences of the adoption of possible alternative strategies. This study aimed to evaluate the growth performance, feed efficiency, carcass traits, and green ham characteristics of heavy pigs raised under the three alternative rearing strategies outlined above, and to compare them with those obtained with the conventional production system.

## 2. Materials and Methods

### 2.1. Pig Housing, Rearing, and Slaughtering

The experiment involved 336 purebred Goland C21 pigs (Gorzagri, Fonzaso, Italy), barrows, and gilts (0.50:0.50), divided into 3 batches of 112 pigs each.

Pigs were members of 68 full-sibling families, generated by mating 13 boars to 67 sows. Besides growth and residual feed efficiency, the breeding goal of the Goland C21 pig line includes traits related to the quality of raw hams [12] and their suitability for dry-curing [13]. All the pigs from a given batch were born in the same week, raised on the same farm, and fed the same commercial diets till their transfer to the experimental station of the University of Padua at 95.0 ± 12.5 kg body weight (BW) and 149 ± 3 days of age. In each of the three batches, the 112 pigs were equally allocated to the four treatment groups, representing the control (C) and three alternative rearing strategies. The 3 batches of pigs entered the experimental period sequentially and experiments were conducted during different seasons (autumn, winter, and spring), avoiding the summer hot environmental temperatures. The duration of the experimental period ranged from 85 to 134 days, depending on the rearing strategy.

Pigs were housed in pens of 5.8 × 3.8 m with fully slatted floors (1.57 m^2^/pig). Each pen was equipped with a single-space electronic feeder (Compident Pig–MLP, Schauer Agrotronic, Prambachkirchen, Austria) programmed to supply each pig with the planned daily amount of feed. The weighting system of each station was calibrated as described in [14]. For each visit and pig, the station recorded the time and date of the feeding event, the time spent eating, and the amount of feed consumed. For the current experiment, the daily feed intake of each pig was computed as the sum of the feed consumed during each visit in the day. Major details about feed distribution, consumption, and feeding behavior measurement were reported previously [15]. Water was accessed freely from nipple drinkers within each pen. The average temperature in the housing rooms was set to 19–22 °C.

Pigs were weighed with an electronic scale at the start and the end of the trial, and at 120 kg BW, in correspondence of the change of feed. At each weighing, backfat depth (BF) was measured with an A-mode ultrasonic device (Renco Lean-Meater series 12, Renco Corporation, Minneapolis, MN, USA). The BF measurements were taken at the last rib at approximately 5.5 to 8.0 cm from the midline, at an increasing distance with increasing BW [16]. The gain in BF depth was computed as a difference between the final and the starting BF depth. When pigs reached the average targeted BW or age, they were subject to fasting for 24 h before being transferred to a commercial abattoir and slaughtered following regulations for commercial practices.

During the trial, one pig died because of gastric torsion, and 10 pigs were moved to the infirmary because of lameness and their data were excluded from the study. A total of 325 records were available for analysis.

### 2.2. Experimental Design

The study, arranged as a split-plot design with treatments and sex within a pen, included 4 treatments, control (C) and 3 groups, representing 3 alternative rearing strategies. The characteristics of the 4 groups of pigs are summarized in Table 1. The C group corresponded to the traditional heavy pig farming system and included pigs fed restrictively medium protein (MP) feeds, with lysine as the first limiting indispensable amino acid (AA). Pigs were slaughtered at about 170 kg SW and 9 months SA. The first alternative rearing strategy aimed to reach 170 kg of SW at the minimum age (younger age, YA). The pigs of this group were fed ad libitum high-protein (HP) feeds, not limiting for indispensable AA content. The second alternative strategy aimed to reach the maximum SW at 9 months SA (greater weight, GW). Pigs of this group were fed ad libitum the same HP feeds of the YA group and they were slaughtered at the same SW as the C pigs. The third alternative treatment (older age, OA) aimed to produce pigs of 170 kg SW with an increased age compared to C. These pigs were fed restrictively as the C group, but with low-protein (LP) feeds containing a low amount of lysine, as the first limiting AA.

In the experimental station, for each of the 3 batches, the 112 pigs were distributed in 8 pens (14 pigs/pen, 2 pens per treatment), with barrows and gilts equally mixed in the same pen. Means and standard deviations of initial BW were similar across the pens. Pigs assigned either to the C group or to one of the treatments (28 pigs per group) were housed in two pens. An across-batch rotation scheme was used to assign treatment groups to pens in different batches so that each treatment was assigned to every pen.

Feeds were manufactured (Progeo Feed Industry, Masone, Reggio Emilia, Italy) using the same batches of feed ingredients. The ingredient composition of early (90 to 120 kg average body weight) and late finishing feeds (over 120 kg body weight) is reported in Table 2.

Feed samples were collected and analyzed to evaluate, before their use in the experiment, the actual nutrient contents. Feed samples (10 samples for each feed collected online to achieve 1-kg feed sample after pooling and mixing) were analyzed for proximate composition [17], starch content [18], and neutral detergent fiber content [19]. The nutrient composition of the feeds, including metabolizable and net energy, crude protein, and AA contents, was computed according to tabular data provided by NRC [20] and is reported in Table 3.

The early finishing HP feed, used in the groups’ YA and GW from 90 to 120 kg BW, was designed to contain non-limiting amounts of indispensable standardized ileal digestible (SID) lysine, methionine, tryptophan, and threonine, according to the NRC recommendation for the 70–100 kg BW range [20]. The SID lysine content of the early finishing MP feed, used in the C group, was 26% lower than that proposed by NRC [20] for the same BW range. Such feed was expected to guarantee an ADG of 0.7 kg/d, with lysine as the first limiting AA. The SID lysine content of the early finishing LP feed, used in the OA group, was limiting, consistent with an ADG of 0.650 kg/d, and was designed to be lower than that used in previous studies, where the limited dietary AA content did not influence growth performance and meat quality [15,21].

Within the treatment groups, the late finishing HP, MP, and LP diets, fed from 120 kg BW on, were formulated to contain about 20–25% less indispensable SID AA than the corresponding HP, MP, and LP feed used in the early finishing period, with lysine as the first limiting AA.

Upon arrival at the experimental station, the amount of feed distributed was estimated based on the average initial BW, and the amount of feed was successively increased weekly without any further adjustment. The amount of feed provided to pigs fed restrictively was increased from 2.3 to 3.0 kg/d for the entire duration of the trial, corresponding to an increase of 57 to 82 g/kg^0.75^ metabolic weight, as per common practice [15].

### 2.3. Slaughter and Evaluation of Carcass and Green Ham Quality

Slaughter and carcass dressing were carried out as described in Schiavon et al. [22]. Hot carcass weight was recorded online, and the lean percentage was estimated by image analysis of the left carcass side (CSB-Image-Meter^®^, CSB-System AG, Geilenkirchen, Germany) according to the EU guidelines [23,24]. Primary cuts (loin with ribs, shoulder, thigh, lard, and belly) were weighed using an electronic scale. Green hams were chilled (0–2 °C) for 24 h, trimmed to obtain the typical round ham shape, and weighed again.

The ham subcutaneous fat depth was measured in the proximity of m. *biceps femoris* (P1) and m. *semimembranosus* (P2) using a caliper and a portable ultrasound system (Aloka SSD 500 equipped with UST-5512 7.5 MHz linear transducer probe, Hitachi Medical Systems S.p.A., Milan, Italy), respectively.

A trained operator scored all left hams as described in Schiavon et al. [15] for round shape (0 = low, to 4 = high, optimum: 1 to 2); visible marbling (0 = absent to 4 = very evident, optimum: 1); fat cover thickness (−4 = very thin to 4 = very thick, optimum: 0 to 1); lean color intensity (−4 = very pale to 4 = very dark, optimum = 0); bicolor, indicating muscles with different color (−4 = absent to 4 = very evident); and veining (0 = absent to 4 = very evident, optimum = 0). A similar scoring grid for these traits has also been reported by Magistrelli et al. [25], and comparable grids are used elsewhere [26,27].

### 2.4. Statistical Analysis

The data were analyzed by the MIXED procedure of SAS (SAS Inst. Inc., Cary, NC, USA) using the following linear model:

y_ijklm_ = µ + RS_i_ + sex_j_ + (RS × sex)_ij_ + batch_k_ + pen(RS × batch)_l:ik_ + e_ijklm_ (1)

where y_ijklm_ was the observed trait, µ was the overall intercept of the model, RS was the fixed effect of the i^th^ rearing strategy (*i* = 1, …, 4), sex was the fixed effect of the j^th^ sex (*j*: 1 = gilts, 2 = barrows), (RS × sex) was the interaction effect between rearing strategy and sex, batch was the random effect of the k^th^ batch (*k* = 1, …, 3), pen was the random effect of the l^th^ pen within the (batch × RS)_ik_ interaction (*l* = 1, 2), and e_ijklm_ was the random residual.

The pen, the batch, and the residuals were assumed to be independently and normally distributed with a mean of zero and variance σ^2^_k_, σ^2^_l_, and σ^2^_e_, respectively. The effect of the rearing strategy was tested on the pen (RS × batch) variance, whereas sex and the rearing strategy × sex interaction were tested on the residual variance. The 3 degrees of freedom due to the rearing strategy were used to run orthogonal contrasts to test the effect of OA, YA, and GW with respect to C.

## 3. Results

### 3.1. Growth Performance

Pigs in the C group were slaughtered at 172 kg SW and 108 days on-feed, corresponding to 257 d SA (Table 4). The ADG was 715 g/d, the gain to feed ratio (feed efficiency) was 0.265, and the mean final backfat depth was 21.9 mm.

The YA pigs were sacrificed at 81 days on feed (27 days earlier than the C). They exhibited greater daily feed intake (*p* < 0.001), ADG (*p* < 0.001), better feed efficiency (*p* = 0.002), higher final backfat depth (*p* = 0.003), and gain in backfat (*p* < 0.001) but lower cumulative feed consumption compared to C (*p* < 0.001).

The GW pigs had greater SW than C (*p* < 0.001) at the same SA. The GW pigs exhibited greater daily and cumulative feed consumption (*p* < 0.001), ADG (*p* < 0.001), final backfat depth (*p* = 0.003), and gain in backfat (*p* < 0.001) than C. Feed efficiency of GW tended to be higher, although not statistically different (*p* = 0.09), than that of C, despite the greater cumulative feed consumption.

The OA pigs required additional 16 days on feed to reach the same SW as C pigs. OA had lower ADG (*p* < 0.001), similar daily feed intakes (*p* = 0.96), and greater cumulative feed consumption (*p* < 0.001), resulting in a lower feed efficiency (*p* < 0.001) than the C_._ There was no significant difference in backfat depth and gain in backfat depth between C and OA at slaughter.

The GW pigs had greater SW than C (*p* < 0.001) at the same SA. The GW pigs exhibited greater daily and cumulative feed consumption (*p* < 0.001), ADG (*p* < 0.001), final backfat depth (*p* = 0.003), and gain in backfat (*p* < 0.001) than C. The feed efficiency of GW tended to be higher, although not statistically different (*p* = 0.09), than that of C, despite the greater cumulative feed consumption.

The OA pigs required an additional 16 days on feed to reach the same SW as C pigs. OA had lower ADG (*p* < 0.001), similar daily feed intakes (*p* = 0.96), and greater cumulative feed consumption (*p* < 0.001), resulting in a lower feed efficiency (*p* < 0.001) than the C_._ There was no significant difference in backfat depth and gain in backfat depth between C and OA at slaughter.

Sex had a significant influence on various traits: barrows had greater initial (*p* = 0.007) and final SW (*p* = 0.034) and a slightly greater daily (*p* = 0.015) and total (*p* = 0.009) feed consumption. Barrows and gilts had similar ADG (*p* = 0.08), but a greater feed efficiency (*p* = 0.009) and a lower final backfat depth (*p* < 0.001) was observed in gilts (Supplementary Materials Table S1). The treatment × sex interaction had minor influences on final SW and cumulative feed consumption (Supplementary Materials Figure S1).

### 3.2. Carcass Traits

The pigs of the YA and C were slaughtered at the same SW, but the YA treatment influenced various carcass traits compared to C (Table 5). YA pigs had similar carcass weights and carcass yields but greater carcass backfat depth (*p* < 0.035) and lower meat percentage (*p* = 0.010) than C. The YA pigs also had greater fat cut yields (*p* < 0.001), namely backfat (*p* < 0.001) and lards (*p* < 0.001), and lower lean cut yields (*p* = 0.011), namely loin with ribs (*p* = 0.009) and shoulder (*p* = 0.002), compared to C. However, the YA treatment did not influence the green and trimmed hams yields nor the trimming losses.

GW treatment increased carcass weight (*p* < 0.001), carcass yield (*p* < 0.001), and carcass backfat depth (*p* < 0.001) but decreased carcass meat percentage (*p* < 0.001) compared to C. The yield of fat cuts increased (*p* < 0.001) for the contribution of both backfat (*p* < 0.001) and lards (*p* < 0.001), whereas the yield of the various lean cuts decreased (*p* < 0.001) compared to C, with the exception of the green hams (*p* = 0.27).

The OA treatment had little or no influence on the major carcass traits, such as weight, yield, backfat depth, and lean meat percentage, compared to C. OA slightly influenced the yield of commercial cuts, being associated with a reduction of the loin with ribs yield (*p* = 0.025).

Sex also had significant effects on carcass traits: gilts had lower carcass weight (*p* = 0.007), carcass yield (*p* = 0.002), and carcass meat percentage (*p* < 0.001) but similar carcass backfat depth, compared to barrows (Supplementary Materials Table S2). The carcass of the gilts also had a greater lean cut yield (*p* < 0.001), namely loins with ribs (*p* < 0.001), shoulders (*p* = 0.050), and green hams (*p* < 0.001), than barrows. The treatment × sex interaction had a limited influence on carcass traits, except for carcass yield (*p* = 0.020), as barrows had greater carcass yield than gilts, in particular in the YA group (Supplementary Materials Figure S2).

The OA treatment had little or no influence on the major carcass traits, such as weight, yield, backfat depth, and lean meat percentage, compared to C. OA slightly influenced the yield of commercial cuts, being associated with a reduction of the loin with ribs yield (*p* = 0.025).

Sex also had significant effects on carcass traits: gilts had lower carcass weight (*p* = 0.007), carcass yield (*p* = 0.002), and carcass meat percentage (*p* < 0.001) but similar carcass backfat depth, compared to barrows (Supplementary Materials Table S2). The carcass of the gilts also had a greater lean cut yield (*p* < 0.001), namely loins with ribs (*p* < 0.001), shoulders (*p* = 0.050), and green hams (*p* < 0.001), than barrows. The treatment × sex interaction had a limited influence on carcass traits, except for carcass yield (*p* = 0.020), as barrows had greater carcass yield than gilts, in particular in the YA group (Supplementary Materials Figure S2).

### 3.3. Green Ham Traits

The YA treatment did not alter the trimmed ham weight but increased the subcutaneous fat covering in the proximity of the biceps femoris muscle (*p* = 0.036), lean color intensity (*p* = 0.030), bicolor scoring (*p* = 0.007), visible marbling (*p* = 0.041), and fat cover thickness score (*p* = 0.011) compared to C (Table 6). The trimmed hams of the GW pigs were heavier than those of C (*p* < 0.001). The hams of the GW pigs also had thicker subcutaneous fat depth in the proximity of the biceps femoris muscle (*p* = 0.04), and a greater round shape score (*p* = 0.038). The OA treatment was associated with a reduction in trimmed ham weight (*p* = 0.005), with an increased thickness of the subcutaneous fat depth in the proximity of the semimembranosus muscle (*p* = 0.002), and with an increased visible marbling score (*p* = 0.001) compared to C. Little or no effects associated with this treatment were observed on other traits.

Sex had little influence on quality traits (Supplementary Materials Table S3), except for the visible marbling score, which was markedly greater in barrows compared to gilts (0.985 vs. 0.636, *p* < 0.001).

## 4. Discussion

### 4.1. Traditional Rearing System

To fulfil the requirements of the current product specifications for Italian PDO dry-cured ham production (i.e., at least 9 months SA and 160 ± 16 kg SW) [6], farmers are forced to apply a restrictive medium-protein feeding regime [15]. However, the degree of restriction is heterogeneous across farms, as feed allowance adopted by the farmers largely depends on their own experience. In this study, we applied a feed restriction consistent with the recommendation of the major Italian companies supplying feed for heavy pigs intended for PDO dry-cured ham production [14,15]. Based on our results, the amount of feed administered to C pigs corresponded approximately to 79% of the average voluntary feed intake, similar to common practices of some regions of Spain [28]. Regarding the growth performance, feed efficiency, and carcass characteristics, our results are similar to those reported in other studies [8,14,21].

The quality of the dry-cured ham largely depends on the characteristics of the green ham before curing, provided the processing is standardized [29,30]. Previous studies suggested that the weight, depths of subcutaneous fat covering, and marbling of green hams are highly correlated with the final quality of the dry-cured product [8,29,31]. In Italy and other countries, the value of the green ham is determined at slaughter based on its weight, subcutaneous fat depths, fat color, and other characteristics. For these reasons, besides growth performance and carcass traits, our study focused on the quality traits of green and trimmed hams.

For the C pigs, the average trimmed ham weight (13.8 kg), subcutaneous fat depth (proximal to the *biceps femoris* muscle, 28.2 mm), and round shape score (1.38, on a 0- to 4-point scale) were within the optimal range. However, their visible marbling (0.57, on a 0- to 4-point scale), fat cover thickness (−0.30, on a −4- to + 4-point scale), and color intensity (−1.32, on a −4- to +4-point scale) were below the optimum. In their review, Čandek-Potokar and Škrlep [3] pointed out that these traits can be influenced by different SA and SW combinations. At the increase of days on feed and SA, pigs tend to become heavier and have larger hams with increased adiposity [3]. On the other hand, a prolonged on-feed period, especially under restricted feeding conditions, could result in increased energy requirements for maintenance, low feed efficiency, and increased nutrient excretion [15].

### 4.2. Decreasing Slaughter Age at Given Bodyweight (Younger Age, YA)

#### 4.2.1. Growth Performance, Feed Efficiency, and Carcass Characteristics

When pigs are kept on diets low in essential AAs, they tend to increase their feed intake, in an attempt to meet the requirement for the deficient nutrients [22,32]. Under such conditions, the increased feed intake causes an extra amount of energy intake, which in turn results in extra fat deposition, also accompanied by a declining weight gain, so that pigs take longer to reach the target weights. Differently, under a non-limiting energy and AAs supply, pigs can express their potential for growth rate and tissue deposition, provided there are no other environmental limiting factors. In our current experiment, the YA and GW groups diets were formulated to be non-limiting in energy and AAs. Thus, it was expected that, under these treatments, the Goland C21 pigs would have approached their potential growth rate and protein and lipid deposition. Knap et al. [33] observed that, due to intensive selection for lean growth and feed efficiency, improved pig genotypes have a reduced potential for fat accretion and, consequently, a reduced voluntary feed intake. Therefore, lean pig genotypes with a low potential for fat accretion exhibit lower feed consumption than pig genotypes with a greater potential for fat accretion [33]. The pigs involved in our study, when exposed to an unlimited amount of feed, evidenced remarkable voluntary feed intake, ADG, and moderate carcass lean meat % (>3200 g/d, >890 g/d, <49%, respectively). The accretion rate (947 g/d) and the feed efficiency (0.285) of YA pigs were in line with those reported for lean pig genotypes of similar BW ranges [2,22,34]. This suggests that the Goland C21 pigs, genetically selected for green ham quality traits [13,27], have a good potential for both lean and fat tissue accretion.

The YA pigs reached the targeted SW 27 days earlier than the C pigs, at 230 days of age. They had higher daily feed consumption, growth rate, and feed efficiency (+23%, +32%, and +7.5%, respectively), and produced fatter carcasses than the C pigs, despite being sacrificed at the same SW. The greater feed efficiency of the YA pigs can be attributed to the shorter rearing period, requiring a lower energy expenditure for maintenance. However, this was partially compensated by greater energy costs due to the increased fat deposition of the YA pigs compared to C, as fat tissue accretion is expensive in energy terms [10]. The economic implication, in terms of feeding costs at the current prices of the feed ingredients used, is that the cost per unit of BW gain of the YA strategy was 7.2% lower than that of the C groups (Table 7).

Lebret et al. [11] subjected pigs to restricted feeding regimes, to increase the SA while maintaining the same SW of the ad libitum control. They found that a 30-day increase in SA had a great influence on carcass and muscle chemical composition, with favorable or unfavorable influences depending on the feeding strategy applied to modify the growth rate. In the cited experiment of Lebret et al. [11], a voluntary feeding regime increased carcass yield, backfat depth, the proportion of fat cuts in the carcass, and intramuscular fat content of both *longissimus dorsi* and *biceps femoris* muscles, and decreased the lean meat percentage and carcass proportions of lean cuts, including ham and loin, compared to a restrictive feeding regime between 30 and 110 kg BW. These responses were consistent with our experiment, despite the difference in the investigated SW range (30–110 kg).

#### 4.2.2. Trimmed Ham Traits

Some authors suggested that it is preferable to slaughter pigs at older ages because of the positive impact of age on the final quality of the dry-cured ham [9]. However, the influence of increasing SA and SW and ham adiposity on the dry-curing aptitude of the ham was often confounded in the literature, as increasing ages were associated with increased BW and ham adiposity [3]. While a greater fat covering reduces dehydration during seasoning, improving ham quality, an earlier SA is thought to increase the dry-curing losses [3,9]. We found that the YA treatment did not change the trimmed ham weight but improved various measures of fat covering depth and the visible marbling score. Hence, our results suggest that the YA strategy might be of interest, as it improves feed efficiency and the associated economic costs, requires less time to finish the pigs, and improves some ham quality traits, compared to the conventional practice. However, apart from effects associated with ham adiposity, improvements of dry-curing aptitude of the ham due to increasing SA also result [3] in lowered moisture of hams, which could reduce the activity of hydrolytic enzymes, decreasing the activity of proteolytic enzymes, and increasing the activity of exopeptidases and lipases, all aspects that can positively affect the quality of the dry-cured ham. Ultimately, it is not clear whether the negative influence exerted by an earlier SA on the dry-curing aptitude can be offset by the favorable increase in ham adiposity. More research on this aspect is needed.

### 4.3. Increasing Slaughter Weight at a Given Age (Greater Weight, GW)

#### 4.3.1. Growth Performance, Feed Efficiency, and Carcass Characteristics

In general, heavy SW is undesired due to poor feed efficiency and increased production costs [1]. However, in dry-cured ham production systems, the decision to slaughter at a given BW is not limited to the feed efficiency of the pigs but is influenced by several factors, such as ham seasoning aptitude and differences in the curing process based on local practices. For this reason, pig growth performance, feed efficiency, and carcass characteristics are variable, making proper comparisons across studies more difficult to perform [35,36,37,38].

It was obvious that pigs subjected to the GW treatment had a greater feed consumption (+20.4%) and better ADG (+24.9%) compared to the C pigs. However, considering the magnitude of the difference between C and GW, it suggests that Goland C21 pigs can exploit remarkable ADG even when the SW is extended to more than 170 kg. Interestingly, the feed efficiency of the GW pigs was similar to that of pigs under the traditional rearing system. Thus, the feeding cost per unit of gained BW of the GW group proved to be equivalent to the traditional C group. Additionally, it was observed that 26% of the carcasses in the GW group were heavier than 168 kg. This corresponds to the new maximum threshold for carcass weight in the proposed revision of the product specifications. Therefore, depending on the pig genotype, it would be necessary to adopt mild feed restriction to limit the full expression of the pig growth potential, while preserving the quality of the green hams.

#### 4.3.2. Trimmed Ham Characteristics

Increased SW is thought to improve ham quality traits, such as weight, fat covering, and marbling [3]. Heavier hams are considered to have better seasoning aptitude, mainly because of lower seasoning losses. Čandek-Potokar and Škrlep [3] indicated that the reason for this is not directly related to ham weight, but it is ascribed to the greater adiposity of the heavier hams. The results of the current research were consistent with these expectations, as the GW treatment increased the ham weight (+10.9%) and the subcutaneous fat depth, in particular in the proximity of the *biceps femoris* muscle (+14.5%), and it is therefore expected to lead to improvements in other qualitative traits of the ham.

We also observed that with the ad libitum feeding regime practiced with the GW treatment, the uniformity of the dressed ham decreased when compared to C. The coefficient of variation for the dressed ham weight was 6.4 and 7.7% for C and GW, respectively. Uniformity commonly refers to the evenness of pig weights at the slaughterhouse but also the size and the weight of the carcass and the retailed cuts [21]. Uniformity is important for the dry-cured ham industry, as the amount of salt used and the duration of salting must be adapted to the weight of the dressed hams [39]. Thus, the mild feed restriction that was suggested above to limit the occurrence of excessive SW is also expected to have some benefits in terms of carcass and ham uniformity, and to prevent excessive ham fat covering that might otherwise occur in some individuals [3,21].

### 4.4. Increased Slaughter Age at a Given BW (Older Age, OA)

The authors of [11,40,41] indicated that a protein restriction, in addition to energy restriction, is a strategy to increase the SA and yielded fatter carcasses, with greater marbling and better meat sensory quality. Such an approach would be interesting, as the use of low-protein diets is also indicated as a strategy to reduce N excretion and the potential environmental impact of pig farming [14]. Previously, studies have shown that low dietary indispensable AA diets fed restrictively to heavy pigs resulted in negligible influence on the ADG, carcass, and green ham quality traits in crossbred pigs [7,15]. Similarly, a dietary protein restriction had a small influence on the chemical profile of seasoned hams produced by pigs of two crossbreeds [41]. These findings suggest that, in those studies, the degree of AA restriction was not limiting for pig growth. Thus, in our present experiment, we applied a stronger reduction in the indispensable AA supply, to compel the pigs to use less energy for lean growth and more for fat accretion. This restriction significantly reduced the ADG of the OA pigs compared to the C pigs and confirms that the lysine supply was a limiting nutrient in the OA group.

Lebret et al. [11] found that a restricted-energy and lysine supply, between 30 and 110 kg BW, increased the feed efficiency, backfat depth, BW, and intramuscular fat content, and decreased the lean meat percentage but did not change ADG. In our study, the OA treatment strongly impaired ADG (−16%) and feed efficiency (−17%) compared to C. In addition, the OA treatment exerted small influences on carcass components, except for a slight, undesired, 3.7% reduction of the trimmed ham weight compared to C. However, the OA treatment was also positively associated with the greatest increase in ham fat covering thickness in correspondence with the *semimembranosus* muscle (+12.5%) and the visible marbling score (+89%) compared to C, in agreement with previous reports [42,43].

The role of fat covering depth in correspondence with the *semimembranosus* muscle on ham quality has been scarcely investigated. In our experiment, the fat covering in this area is much thinner than that located close to the *biceps femoris* muscle. A sufficient subcutaneous fat layer is necessary to prevent rapid desiccation, which would cause the formation of a crust on the ham surface, and to reduce processing water losses [44,45]. Hams with thinner fat layers are also expected to have a greater NaCl content because of the negative correlation between fat thickness, seasoning losses, and salt content [3,46]. Nonetheless, despite being undesired due to health concerns, a high salt content in cured hams affects the product flavor, chemical, and biochemical processes, such as proteolysis, lipolysis, and lipid oxidation [3,44,46]. Thus, a greater thickness of the fat covering in this area is desired and the trait is included in the selection index of the Goland C21 sire line [13].

Overall, the OA treatment was inefficient, from both a nutritional and economic point of view. The decreased ADG and the increased time required to reach the target slaughter weight resulted in a marked reduction of feed efficiency with little benefits in terms of improved carcass and ham characteristics.

### 4.5. Sex and Treatment × Sex Interaction

Sex influenced growth performance and carcass characteristics. The effects of sex agreed with previous studies, as the barrows consumed more feed, were less efficient, their carcass was fatter, and their hams were characterized by greater marbling than gilts [2,46,47]. In our previous studies conducted on the same pig genetic line, kept under a restricted feeding regime, some differences due to the sex were observed but they were of a small magnitude [14]. With pigs kept on a voluntary feeding regime, we did expect greater differences between sexes, because of a better possibility to exploit the genetic propensity for the growth of various body components. Gilts had a carcass yield >81% only in the GW group, while barrows had carcass yield >81% in all the *ad libitum* treatments YA and GW. This reflects a greater propensity for fat accretion at earlier ages of barrows compared to gilts. However, in the current study, the magnitude of the differences induced by the treatment × sex interaction was small, and the statistical level of significance was rarely reached. This result suggests that the gilts and barrows of the Goland C21 pig genotype have similar responses when exposed to the different treatments of the current study.

## 5. Conclusions

Despite the positive effect of the OA strategy on visible marbling and subcutaneous fat depth proximal to the *semimembranosus* muscle, this strategy was found to be inefficient as it impairs growth and feed efficiency and increases the production costs, with little influence on carcass composition, and with a reduction in ham size compared to the conventional practice.

The best rearing strategy, from an economic point of view, would be the YA strategy, as it permits anticipation of the slaughter by about 27 days earlier, with the highest improvements in ADG, feed efficiency, and ham adiposity. However, the use of this strategy should be applied with caution, as more research is required to clarify whether increased ham adiposity can compensate for the negative effects of younger slaughter age on dry-curing aptitude.

The GW strategy was associated with increased feed consumption, ADG, carcass, and ham weight, with an improvement in some ham quality indices compared to C. Due to its feed efficiency and competitive feeding costs, the GW strategy could be used in place of the conventional practice. However, the adoption of this strategy would be associated with the risk of an increased proportion of carcasses that weigh more than the maximum threshold indicated by the product specifications (168 kg). In such a case, to avoid a depreciation of the value of the carcass, mild feed restriction should be applied depending on the pigs’ growth potential.

## Figures and Tables

**Table 1:** Characteristics of the experimental groups raised according to the traditional (control, C), and three alternative rearing strategies (younger age, YA; greater weight, GW; and older age, OA) ^a^.

	Rearing Strategy			
Item	Control (C)	YA	GW	OA
Weight on arrival, kg	95 ± 13	95 ± 13	95 ± 12	95 ± 12
Age on arrival, d	149 ± 3	149 ± 3	149 ± 3	149 ± 3
Target weight at slaughter, kg	170	170	>170	170
Target age at slaughter, d	270	<270	270	>270
Feeding regime	Restricted	Ad libitum	Ad libitum	Restricted
Protein content in early finishing feed ^b^	Medium	High	High	Low
Protein content in late finishing feed ^c^	Medium	High	High	Low

^a^ C system: 160 ± 16 kg slaughter weight (SW) and 9 months slaughter age (SA); YA = minimum SA at 160 ± 16 kg target SW; GW = maximum SW at 9 months SA, and OA = increased SA at 160 ± 16 kg target SW. ^b^ Early finishing feed was administered from 90 to 120 kg. ^c^ Late finishing feed was administered from 120 kg onward.

**Table 2:** Ingredient composition (g/kg as-fed) of early (90 to 120 kg average body weight) and late finishing feeds (over 120 kg body weight).

	Early Finishing Feeds			Late Finishing Feeds		
Ingredient	High Protein	Medium Protein	Low Protein	High Protein	Medium Protein	Low Protein
Corn grain	361.3	350.0	390.0	398.3	398.3	398.8
Wheat grain	240.0	270.0	260.0	237.8	237.3	237.5
Barley grain	100.0	100.0	100.0	100.0	100.0	100.0
Soybean meal 48% (solv. ex.)	196.0	85.0	38.0	143.0	56.0	18.3
Wheat bran	26.5	87.5	85.3	7.5	57.5	62.5
Wheat middlings	-	20.0	30.0	40.0	67.5	90.0
Cane molasses	20.0	20.0	20.2	22.5	22.5	22.5
Lard	20.0	21.6	21.0	20.0	20.0	20.0
Dried sugar beet pulp	-	10.0	20.0	-	10.0	20.5
Calcium carbonate	15.0	15.0	15.0	13.0	13.0	13.0
Dicalcium phosphate	4.5	4.5	4.5	2.0	2.0	2.0
Sodium chloride	3.0	3.0	3.0	3.0	3.0	3.0
Sodium bicarbonate	2.5	2.5	2.5	2.5	2.5	2.5
Vitamin and mineral premix ^a^	2.0	2.0	2.0	2.0	2.0	2.0
Grapeseed meal	7.5	7.5	7.5	7.5	7.5	7.5
Choline, liquid, 75% ^b^	0.5	-	-	-	-	-
L-Lysine ^c^	1.0	1.4	0.65	-	1.0	1.0
DL-Methionine ^d^	0.2	-	-	-	-	-

^a^ Providing per kilogram of feed: vitamin A, 8000 IU; vitamin D3, 1200 IU; vitamin E, 8 mg; Vitamin B7, 0.08 mg; vitamin B12, 0.012 mg; niacin, 16.0 mg; biotin, 8 mg; iron, 170 mg; zinc, 117 mg; copper, 14 mg; cobalt, 0.11 mg; iodine, 0.06 mg; manganese, 65 mg; magnesium, 0.14 mg; selenium 10 mg. ^b^ Choline liquid 75% (Methodo Chemicals, 42017 Novellara, RE, Italy). ^c^ L-Lysine Monoclohydrate, 98.5% pure, 78% L-Lysine (Methodo Chemicals, 42017 Novellara, RE, Italy). ^d^ DL-Methionine, 98% pure min. (Methodo Chemicals, 42017 Novellara, RE, Italy).

**Table 3:** Nutrient content (g/kg as-fed, unless otherwise indicated) of early (90 to 120 kg average body weight) and late finishing feeds (over 120 kg average body weight) ^a^.

	Early Finishing Feeds			Late Finishing Feeds		
Items	High Protein	Medium Protein	Low Protein	High Protein	Medium Protein	Low Protein
Analyzed nutrient composition						
DM	906	904	904	906	902	904
CP (N × 6.25)	162	128	113	138	119	104
Starch	413	460	488	483	470	490
Ether extract	43	46	44	48	50	48
NDF	131	138	141	118	132	134
Ash	48	47	48	42	41	41
Calculated nutrient composition ^b^						
ME, MJ/kg	13.4	13.2	13.2	13.4	13.2	13.1
NE, MJ/kg	10.0	10.0	10.1	10.1	10.0	9.9
CP (N × 6.25)	162	128	109	142	116	103
Starch	424	449	470	454	470	477
Ether extract	44	47	47	46	47	47
Linoleic acid	14	15	16	15	16	17
Lysine	8.3	6.2	4.6	6.9	5.2	3.6
Methionine	2.7	2.0	1.9	2.2	1.9	1.7
Threonine	5.7	4.3	3.6	5.1	4.0	3.5
Triptophan	2.0	1.5	1.3	1.6	1.3	1.0
Tyrosine	5.3	4.1	3.5	5.0	3.9	3.4

^a^ Analytical result are the average of 3 independent replications. ^b^ Computed based on the NRC (2012) tabular values of each feed ingredient.

**Table 4:** Growth performance of heavy pigs raised according to the traditional rearing system (control, C), and three alternative strategies (younger age, YA; greater weight, GW; and older age, OA) ^a^.

	Rearing Strategy					*p* Values		
Item	C	YA	GW	OA	SEM ^b^	C vs. YA	C vs. GW	C vs. OA
Animals, n	83	77	82	83	-	-	-	-
Days on feed ^c^, d	108	81	108	124	-	-	-	-
Initial bodyweight, kg	95.1	95.5	95.7	95.0	6.1	0.78	0.69	0.93
Slaughter weight, kg	172.7	172.3	192.9	169.3	1.5	0.81	<0.001	0.11
Daily feed consumption, g/d	2694	3310	3245	2697	42	<0.001	<0.001	0.96
Cumulative feed consumption, kg/pig	293	267	353	334	24	<0.001	<0.001	<0.001
Average daily gain, g/d	715	947	893	598	22	<0.001	<0.001	<0.001
Gain to feed ratio	0.265	0.285	0.275	0.221	0.007	0.002	0.09	<0.001
Backfat depth, mm								
initial	12.2	11.9	12.2	11.8	1.89	0.41	0.95	0.26
at slaughter	21.9	24.8	26.0	22.9	0.91	0.026	0.003	0.26
Gain in backfat depth, mm	9.7	12.9	13.9	11.1	1.68	0.004	<0.001	0.08

^a^ C system: 160 ± 16 kg slaughter weight (SW) and 9 months slaughter age (SA); YA = minimum SA at 160 ± 16 kg target SW; GW = maximum SW at 9 months SA, and OA = increased SA at 160 ± 16 kg target SW. ^b^ SEM: pooled standard error of the mean, n = 325. ^c^ At the start of the experiment, pigs were 149 ± 3 d old.

**Table 5:** Carcass traits of heavy pigs raised according to the traditional rearing system (control, C), and three alternative strategies (younger age, YA; greater weight, GW; and older age, OA) ^a^.

	Rearing Strategy						*p*-Values	
Item	C	YA	GW	OA	SEM ^b^	C vs. YA	C vs. GW	C vs. OA
Carcass weight, kg	140	141	160	138	1.4	0.65	<0.001	0.22
Carcass yield, %	81.2	81.8	82.9	81.5	0.27	0.10	<0.001	0.38
Backfat depth ^c^, mm	37.9	41.1	45.6	38.5	0.96	0.035	<0.001	0.71
Lean meat, %	50.8	48.8	47.4	50.4	0.50	0.010	<0.001	0.53
Commercial cuts yield, g/kg carcass								
Fat cuts	189	211	209	194	2.6	<0.001	<0.001	0.20
Backfat	122	129	130	123	2.2	<0.001	<0.001	0.57
Lard	67	82	79	71	2.5	<0.001	<0.004	0.28
Lean cuts	529	517	513	521	4.6	0.011	0.001	0.06
Loin with rib	154	148	145	149	1.7	0.009	<0.001	0.025
Shoulder	136	129	130	136	2.7	0.002	0.005	0.83
Green ham	240	240	238	236	1.4	0.86	0.27	0.05
Trimmed ham ^d^	197	195	191	193	1.9	0.27	0.008	0.06
Trimming ham loss ^d^	43.2	45.6	46.6	42.9	1.2	0.07	0.013	0.82

^a^ C system: 160 ± 16 kg slaughter weight (SW) and 9 months slaughter age (SA); YA = minimum SA at 160 ± 16 kg SW; GW = maximum SW at 9 months SA, and OA = increased SA at 160 ± 16 kg SW. ^b^ SEM: pooled standard error of the mean, n = 325. ^c^ Average of backfat depth measured with a caliper at the points of maximum depth at the shoulder and the loin. ^d^ Trimming was performed at the slaughterhouse the day after slaughtering.

**Table 6:** Green and trimmed ham characteristics of heavy pigs raised according to the traditional rearing system (control, C), and three alternative strategies (younger age, YA; greater weight, GW; and older age, OA) ^a^.

	Rearing Strategy					*p*-Values		
Item	C	YA	GW	OA	SEM ^b^	C vs. YA	C vs. GW	C vs. OA
Trimmed ham weight, kg	13.8	13.7	15.3	13.3	0.19	0.55	<0.001	0.005
Subcutaneous fat depth P1, mm ^c^	28.2	32.4	32.3	28.7	2.57	0.036	0.040	0.78
Subcutaneous fat depth P2, mm ^d^	6.4	6.8	6.8	7.2	0.3	0.08	0.06	0.002
Round shape (0 to 4) ^e^	1.38	1.72	1.82	1.31	1.20	0.20	0.038	0.71
Visible marbling (0 to 4) ^f^	0.57	0.87	0.73	1.08	0.38	0.041	0.26	0.001
Fat cover thickness (−4 to 4) ^g^	−0.30	0.98	0.53	0.18	0.32	0.011	0.08	0.30
Lean color intensity (−4 to 4) ^h^	−1.32	−0.54	−0.73	−0.99	0.49	0.030	0.09	0.33
Bicolor (−4 to 4) ^i^	1.08	2.28	1.38	1.56	0.33	0.007	0.40	0.29
Veining (0 to 4) ^l^	1.13	1.57	1.48	1.15	0.13	0.14	0.36	0.16

^a^ C system: 160 ± 16 kg slaughter weight (SW) and 9 months slaughter age (SA); YA = minimum SA at 160 ± 16 kg SW; GW = maximum SW at 9 months SA, and OA = increased SA at 160 ± 16 kg SW. ^b^ SEM: Standard error of means, n = 325. ^c^ Measured in the proximity of m. *biceps femoris* (the higher the better). ^d^ Point of minimum fat depth, measured in proximity of m. *semimembranosus* (the higher the better). ^e^ Round shape (0 = low, 4 = high, optimum: 1 to 2). ^f^ Visible marbling (0 = absent, 4 = very evident, optimum = 1). ^g^ Fat cover thickness (−4 = very thin, 4 = very thick, optimum: 0 to 1). ^h^ Lean color intensity (−4 = very pale, 4 = very dark, optimum = 0). ^i^ Bicolor (0 = absent, 4 = very evident, optimum = 0). ^l^ Veining (0 = absent, 4 = very evident, optimum = 0).

**Table 7:** Feeding costs of pigs raised according to the traditional (control, C), and three alternative strategies (younger age, YA; greater weight, GW; and older age, OA) ^a^.

	Rearing Strategy			
Item	C	YA	GW	OA
Feed price, euro/ton as fed	338	348	348	327
Feeding costs:				
Euro/pig produced	106.2	97.79	130.5	119.3
Euro/kg BW gain	1.369	1.273	1.343	1.606

^a^ C system: 160 ± 16 kg slaughter weight (SW) and 9 months slaughter age (SA); YA = minimum SA at 160 ± 16 kg SW; GW = maximum SW at 9 months SA, and OA = increased SA at 160 ± 16 kg SW.

## Data Availability

The data supporting the findings of this study are available from Gorzagri s.s., but restrictions apply to the availability of these data, which were used under license for the current study and are not publicly available. Data are however available from the authors upon reasonable request and with permission of Gorzagri s.s.

## Supplementary Materials

The following are available online at https://www.mdpi.com/article/10.3390/ani11082447/s1, Table S1: Growth performance of gilts and barrows raised according to the traditional rearing system (Control, C), and three alternative strategies (Age-, Weight+ and Age+) (Younger Age, YA; Greater Weight, GW; and Older Age, OA) ^a^, Table S2: Carcass traits of gilts and barrows raised according to the traditional rearing system (Control, C), and three alternative strategies (Younger Age, YA; Greater Weight, GW; and Older Age, OA) ^a^, Table S3: Green ham characteristics of gilts and barrows raised according to the traditional rearing system (Control, C), and three alternative strategies (Younger Age, YA; Greater Weight, GW; and Older Age, OA) ^a^, Figure S1: Influence of the sex × rearing strategy interaction (least square means ± standard deviation; sex × rearing Scheme 0. on cumulative feed consumption of heavy pigs raised according to the traditional rearing system (Control, C), and three alternative strategies (Younger Age, YA; Greater Weight, GW; and Older Age, OA). [C system: 160 ± 16 kg slaughter weight (SW) and 9 months slaughter age (SA); YA = minimum SA at 160 ± 16 kg SW; GW = maximum SW at 9 months SA, and OA = increased SA (>9 months) at 160 ± 16 kg SW; n = 325], Figure S2: Influence of the sex × rearing strategy interaction (least square means ± standard deviation; sex × rearing Scheme 0. on cumulative feed consumption of heavy pigs raised according to the traditional rearing system (Control, C), and three alternative strategies (Younger Age, YA; Greater Weight, GW; and Older Age, OA). [C system: 160 ± 16 kg slaughter weight (SW) and 9 months slaughter age (SA); YA = minimum SA at 160 ± 16 kg SW; GW = maximum SW at 9 months SA, and OA = increased SA (>9 months) at 160 ± 16 kg SW; n = 325].
