# Supplementary material for: The Implications of Changing Age and Weight at Slaughter of Heavy Pigs on Carcass and Green Ham Quality Traits
Source: Animals (Basel). 2021 Aug 20;11(8):2447. doi: 10.3390/ani11082447 (PMC8388713; doi:10.3390/ani11082447)
Supplement: Supplementary file 1 [file animals-11-02447-s001.zip › animals-1316994-supplementary.pdf]

animals

Supplementary Tables and Figures

Table S1. Growth performance of gilts and barrows raised according to the traditional rearing system (Control, C), and three alternative strategies (Age-, Weight+ and Age+) (Younger Age, YA; Greater Weight, GW; and Older Age, OA) [a].

	Sex			*p*-values	
**Item**	**Gilts**	**Barrows**	**SEM** [b]	**Sex**	**Sex × Rearing Strategy**
Body weight, kg					
Initial	94.0	96.7	6.1	0.007	0.37
Final	175.3	178.3	1.0	0.034	0.030
Feed consumption, g/d	2949	3024	42	0.015	0.06
Cumulative feed consumption, kg	308	316	24.2	0.009	0.024
Average daily gain, g/d	787	789	16.3	0.85	0.08
Gain to feed ratio	0.265	0.259	0.006	0.009	0.64
Backfat depth, mm	23.3	24.5	0.78	0.002	0.75
Gain in backfat, mm	11.9	11.9	1.62	0.81	0.57

[a] C_{170} system: 160 ± 16 kg slaughter weight (SW) and 9 months slaughter age (SA); YA = minimum SA at 160 ± 16 kg SW; GW = maximum SW at 9 months SA, and OA = increased SA at 160 ± 16 kg SW. [b] SEM: pooled standard error of the mean, n = 325.

Animals **2021**, *11*, 2447. https://doi.org/10.3390/ani11082447 www.mdpi.com/journal/animals

Table S2. Carcass traits of gilts and barrows raised according to the traditional rearing system (Control, C), and three alternative strategies (Younger Age, YA; Greater Weight, GW; and Older Age, OA) [a].

Item	Sex: Gilts	Sex: Barrows	SEM [b]	*p*-values: Sex	*p*-values: Sex × Rearing Strategy
Carcass weight, kg	143	146	1.0	0.007	0.07
Carcass yield, %	81.6	82.1	0.17	0.002	0.020
Backfat depth [c], mm	40.4	41.1	0.57	0.28	0.71
Lean meat, %	50.4	48.2	0.36	<0.001	0.27
Commercial cuts yield, g/kg carcass					
Fat cuts	199	202	1.6	0.04	0.24
Backfat	126	125	2.0	0.18	0.62
Lards	73	77	1.4	<0.001	0.14
Lean cuts	526	515	4.0	<0.001	0.14
Loin with ribs	152	146	1.4	<0.001	0.20
Shoulder	133	132	2.5	0.050	0.58
Green hams	240	237	0.87	<0.001	0.14
Trimmed hams [d]	196	192	1.57	<0.001	0.19
Trimming ham losses [e]	45	45	1.92	0.93	0.37

[a] C system: 160 ± 16 kg slaughter weight (SW) and 9 months slaughter age (SA); YA = minimum SA at 160 ± 16 kg SW; GW = maximum SW at 9 months SA, and OA = increased SA at 160 ± 16 kg SW. [b] SEM: pooled standard error of the mean, n = 325. [c] Average of backfat depth measured with a caliper at the points of maximum depth at the shoulder and the loin. [d] Trimming performed at the slaughterhouse the day after slaughtering. [e] Trimming ham losses were computed as the difference between the green ham and the trimmed ham weights.

Table S3. Green ham characteristics of gilts and barrows raised according to the traditional rearing system (Control, C), and three alternative strategies (Younger Age, YA; Greater Weight, GW; and Older Age, OA) [a].

Item	Sex		SEM [b]	*p*-values	
	Gilts	Barrows		Sex	Sex × Rearing System
Trimmed ham weight, kg	13.9	14.0	0.17	0.59	0.08
Subcutaneous fat depth P1, mm [c]	22.7	24.13	2.34	0.06	0.34
Subcutaneous fat depth P2, mm [d]	6.74	6.82	0.23	0.51	0.63
Round shape (0 to 4) [e]	1.58	1.54	0.16	0.69	0.49
Visible marbling (0 to 4) [f]	0.64	0.98	0.06	<0.001	0.83
Fat cover thickness (-4 to 4) [g]	0.21	0.48	0.18	0.07	0.40
Lean colour intensity (- 4 to 4) [h]	-0.97	-0.82	0.45	0.32	0.71
Bicolor (-4 to 4) [i]	1.64	1.51	0.25	0.38	0.96
Veining (0 to 4) [l]	1.32	1.45	0.11	0.19	0.94

[a] C system: 160 ± 16 kg slaughter weight (SW) and 9 months slaughter age (SA); YA = minimum SA at 160 ± 16 kg SW; GW = maximum SW at 9 months SA, and OA = increased SA at 160 ± 16 kg SW. [b] SEM: pooled standard error of the mean, n = 325. [c] Ham subcutaneous fat depth measured in proximity of m. *biceps femoris* with a calliper (the hgher the better). [d] Ham subcutaneous fat depth measured at the point of minimum depth, in proximity of m. *semimembranosus* with a portable ultrasound system (the higher the better). [e] Round shape (0 = low, 4 = high, optimum: 1 to 2). [f] Visible marbling (0 = absent, 4 = very evident, optimum = 1). [g] Fat cover thickness (−4 = very thin, 4 = very thick, optimum: 0 - 1). [h] Lean color intensity (−4 = very pale, 4 = very dark, optimum = 0). [i] Bicolor (0 = absent, 4 = very evident, optimum = 0). [l] Veining (0 = absent, 4 = very evident, optimum = 0).

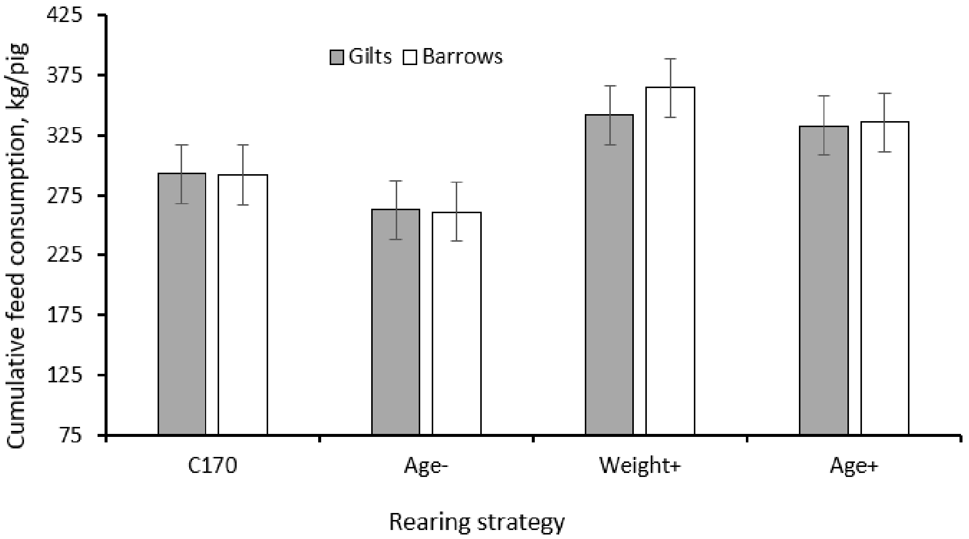

Figure S1. Influence of the sex × rearing strategy interaction (least square means ± standard deviation; sex × rearing Scheme 0. on cumulative feed consumption of heavy pigs raised according to the traditional rearing system (Control, C), and three alternative strategies (Younger Age, YA; Greater Weight, GW; and Older Age, OA). [C system: 160 ± 16 kg slaughter weight (SW) and 9 months slaughter age (SA); YA = minimum SA at 160 ± 16 kg SW; GW = maximum SW at 9 months SA, and OA = increased SA (> 9 months) at 160 ± 16 kg SW; *n* = 325].

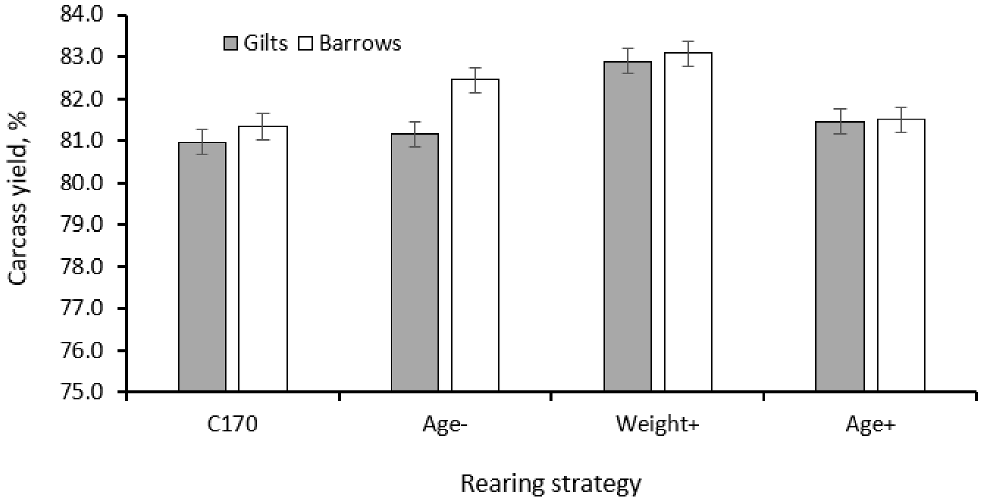

Figure S2. Influence of the sex × rearing strategy interaction (least square means ± standard deviation; sex × rearing strategy interaction p =0.020) on carcass yield of heavy pigs raised according to the traditional rearing system (Control, C), and three alternative strategies (Younger Age, YA; Greater Weight, GW; and Older Age, OA). [C system: 160 ± 16 kg slaughter weight (SW) and 9 months slaughter age (SA); YA = minimum SA at 160 ± 16 kg SW; GW = maximum SW at 9 months SA, and OA = increased SA (> 9 months) at 160 ± 16 kg SW; *n* = 325].
